# Advantages of visualisations to evaluate and communicate adverse event information in randomised controlled trials

**DOI:** 10.1186/s13063-020-04903-0

**Published:** 2020-12-22

**Authors:** Victoria Cornelius, Suzie Cro, Rachel Phillips

**Affiliations:** Imperial Clinical Trials Unit, Imperial College London, 1st Floor Stadium House, 68 Wood Lane, London, W12 7RH UK

**Keywords:** Randomised controlled trials, Adverse events, Adverse reactions, Harms, Visualisation, Graphics, Reporting, Data analysis

## Abstract

**Background:**

Randomised controlled trials (RCTs) provide valuable information and inform the development of harm profiles of new treatments. Harms are typically assessed through the collection of adverse events (AEs). Despite AEs being routine outcomes collected in trials, analysis and reporting of AEs in journal articles are continually shown to be suboptimal. One key challenge is the large volume of AEs, which can make evaluation and communication problematic. Prominent practice is to report frequency tables of AEs by arm. Visual displays offer an effective solution to assess and communicate complex information; however, they are rarely used and there is a lack of practical guidance on what and how to visually display complex AE data.

**Methods:**

In this article, we demonstrate the use of two plots identified to be beneficial for wide use in RCTs, since both can display multiple AEs and are suitable to display point estimates for binary, count, or time-to-event AE data: the volcano and dot plots. We compare and contrast the use of data visualisations against traditional frequency table reporting, using published AE information in two placebo-controlled trials, of remdesivir for COVID-19 and GDNF for Parkinson disease. We introduce statistical programmes for implementation in Stata.

**Results/case study:**

Visualisations of AEs in the COVID-19 trial communicated a risk profile for remdesivir which differed from the main message in the published authors’ conclusion. In the Parkinson’s disease trial of GDNF, the visualisation provided immediate communication of harm signals, which had otherwise been contained within lengthy descriptive text and tables. Asymmetry in the volcano plot helped flag extreme events that were less obvious from review of the frequency table and dot plot. The dot plot allowed a more comprehensive representation by means of a more detailed summary.

**Conclusions:**

Visualisations can better support investigators to assimilate large volumes of data and enable improved informal between-arm comparisons compared to tables. We endorse increased uptake for use in trial publications. Care in construction of visual displays needs to be taken as there can be potential to overemphasise treatment effects in some circumstances.

**Supplementary Information:**

The online version contains supplementary material available at 10.1186/s13063-020-04903-0.

## Background

When evaluating a new treatment in a randomised controlled trial (RCT), it is well accepted that potential harms should be monitored alongside any potential benefit. Harms are assessed through the collection of adverse events (AEs) that occur during the trial period. Information on AEs is collected from several sources within an RCT and typically include spontaneous reports from participants and clinical observations [1]. AEs are most often recorded as a binary variable, i.e. an event that does or does not occur to a participant during the study follow-up period. As well as spontaneous reporting of events by participants and clinicians, many trials include clinical and biological monitoring at regular intervals as a way to screen for harm. Monitored events are frequently continuous in nature but are often dichotomised into binary or categorical variables for the analysis. For binary AEs of special interest, these are sometimes examined in a time-to-event framework.

Some of these AEs will have occurred to the participants regardless of their involvement in the trial and are therefore unrelated to the treatment, and those that are related to treatment are termed adverse reactions (ARs) [2, 3]. Trying to ascertain which AEs are related to the treatment (and are ARs) is difficult. While methods to analyse efficacy outcomes in RCTs have been well developed, the analysis of AEs encounters greater challenges due to limited sample size in trials, expected low event rates, multiple outcomes, and the variety and complexity of AE data [4].

How to analyse, summarise, and present large numbers of AEs collected in a trial is a primary challenge. Prominent ‘analysis’ practice in published RCT articles is the use of contingency tables to present frequencies by arm [5, 6]. The between-arm event rates in the table are either subjectively assessed by an investigator, or inappropriate hypothesis testing is employed, often with literal interpretation of a *p* value < 0.05 as a way to identify an AR [5–7]. Hypothesis testing is not recommended as statistical power is limited when events are binary or counts and multiplicity of testing is an issue [8, 9].

It can be problematic to evaluate (often lengthy) lists of AEs in tables. Visual representation of this information can provide an immediate and informal way to effectively communicate the same information, and facilitate the identification of harm signals. In 2016, a collaboration of industry experts and journal editors undertook a consensus and published recommendations to improve AE reporting in trial publications [10]. The recommendations included presenting a summary of clinically important events, reporting important minimum numeric information, and, in line with the CONSORT harms extension [11] and recommendations from the Safety Planning, Evaluation and Reporting Team (SPERT) [12], a proposal to encourage the use of graphical displays to visualise AE data. While graphical approaches have been promoted for the presentation and analysis of AEs [13–15], the use of plots to display AE data in journal articles is uncommon. A recent review of phase II/III trials found only 12% of articles included a graph of any harm outcomes, of these 5% included a graph of multiple binary AE outcomes and the other 7% included graphs of continuous outcomes [6]. There remains a lack of information and guidance on what and how to visually display complex AE data.

In this article, we critically appraise the presentation and description of AEs reported in two recently published RCTs of drug interventions and illustrate how the same information can be presented graphically using two newly develop statistical commands. We contrast the two plots (volcano and dot) with the originally published tables to demonstrate the strength and limitations of each way to display the information.

## Methods

### Data visualisations

We include two plots that were identified in a recent AE methodology review (publication in press, pre-print available [16]). They were chosen as they were assessed to be particularly beneficial for wide use in published reports of RCTs in journal articles since both display multiple AEs, and are versatile, being able to display point estimates for binary, count, or time-to-event AE data. Statistical programmes for implementation in Stata were developed to implement these visual methods [17, 18].

The first is the *volcano plot* proposed by Zink et al. in 2013 [19]. The volcano plot displays a between-arm summary statistic on the *x*-axis, against the log10-transformed *p* value from a test of the researcher’s choice on the *y*-axis. The summary statistic plotted can be the absolute risk difference, or alternatively a relative measure such as the risk ratio, odds ratio, or the incidence rate ratio. This plot can be produced in Stata using the aevolcano [17] or aevolcs command dependent on the structure of the dataset (see example datasets in supplementary material).

The relevant Stata command and associated help files can be freely installed by typing into the Stata command line:

- ssc install aevolcano

or it can be downloaded at https://ideas.repec.org/c/boc/bocode/s458736.html.

For binary AE data, the Stata command calculates the *p* value from the Fisher’s exact test or the chi-squared test as requested by the user. The command will incorporate a correction when fitting odds ratios or risk ratios if there are zero events in one of the treatment arms by adding half an event to each arm (numerator and denominator). This is a commonly used continuity correction but has been shown to be inferior when used for meta-analysis models on rare events as it will affect the variance and therefore its weight in the model [20]. While this common correction has been employed here, alternative corrections may want to be considered. To adjust for multiple testing, Zink et al. suggest incorporation of a multiplicity adjustment such as the false discovery rate (FDR) as proposed by Mehrotra and Adewale when calculating *p* values [21]. The FDR adjustment can be undertaken by grouping individual AEs into aggregated levels such as terms for body systems (e.g. respiratory, neurological, and dermatological). It then uses this structure to make a multiplicity adjustment. With suitable datasets, the FDR adjustment can be used within the aevolcano and aevolcs command by adding the applicable options.

Under a null hypothesis of no difference, the expected shape of the plot would be symmetrical around the value of no difference for the summary statistic used. The volcano plot can provide a way to immediately identify outlying events for further investigation by means of an asymmetrical plot. Any asymmetry should not be overinterpreted as an ADR as in practice, when there are low numbers of adverse events included or when there are differing incidence rates, it will not be uncommon to obtain an asymmetrical plot. The plot should be used as a means to identify ‘signals’ of ADRs taking into consideration the role that the number of AEs included and the heterogeneity in incidence rates have in this visual impression.

The second plot is the *dot plot*, first proposed by Amit et al. for AE data in 2008 [13]. The dot plot displays the percentage of participants experiencing an AE (each event labelled on the *y*-axis) in each treatment arm on the left hand side of the plot and a relative measure, such as the relative risk, with corresponding 95% confidence interval (CI), on the right hand side. An indication of the precision around estimates is particularly important when the event rate is low. We propose an adaption to the original plot proposed by Amit et al. [13] and include a display of the number of participants with each AE by arm and the number of events can also be added if wanted. This can be implemented by adding the relevant options to the Stata command. Such additional information may be desirable in situations such as when the event rate is low. This plot can be produced in Stata by using the aedot or aedots command [18] dependent on the structure of the data (example datasets can be found in the supplementary material).

The relevant Stata command and associated files can be installed using the Stata ssc install command, typing into the Stata command line:

- ssc install aedot

or downloaded at https://ideas.repec.org/c/boc/bocode/s458735.html.

If zero events are experienced in either treatment arm, the Stata command adds half an event to each arm (numerator and denominator) to calculate the relative risk, standard error, and 95% CI. This does not affect the percentages presented or calculation of the risk difference. The calculation of the 95% CI uses the normal approximation.

The dot plot is a useful, space-efficient, visual alternative to the traditional two-by-two table AE data is typically displayed in, providing a clear and concise summary of the overall burden of harm with a measure of precision for the relative point estimate.

In this article, two recently published high-profile drug intervention trials are used to demonstrate the implementation of these plots [22, 23]. We appraised the reported AE results and used the volcano and dot plots to demonstrate the impact of visualising information typically displayed in tables and their utility for communication and interpretation of AE results. Aggregate data was extracted from the tables in the published articles. We provide the extracted data along with example Stata code to demonstrate implementation (supplementary material).

## Results

### Case study 1: Remdesivir in adults with severe COVID-19: a multicentre randomised, double-blind, placebo-controlled trial

#### Study design

Wang et al. undertook a randomised, multicentre, placebo-controlled trial of remdesivir in adults admitted to hospital with severe COVID-19 infections in Wuhan, China [22]. Remdesivir is a broad antiviral drug with evidence of clinical effectiveness in Middle East respiratory syndrome (MERS) infections. The trial was originally powered to detect a change in time to clinical improvement within 28 days (hazard ratio of 1.4) and required 325 events (participants achieving clinical improvement) with 80% power and one-sided type one error 2.5% (recruitment needed *n* ≈ 453 participants) in a 2:1 ratio. The trial was stopped early by the Data Monitoring Committee after recruiting 237 patients (158 to remdesivir, 79 to placebo). The decision was stated to be due to a higher proportion of participants discontinuing from the study treatment due to AEs (12% versus 5%), alongside consideration of the improved control of the COVID-19 outbreak in Wuhan.

In the study, there was daily monitoring of AEs and screening of AEs using clinical laboratory tests on days 1, 3, 7, and 10; 12-lead electrocardiogram on days 1 and 14; and daily vital signs.

#### Results

**Fig. 1:**
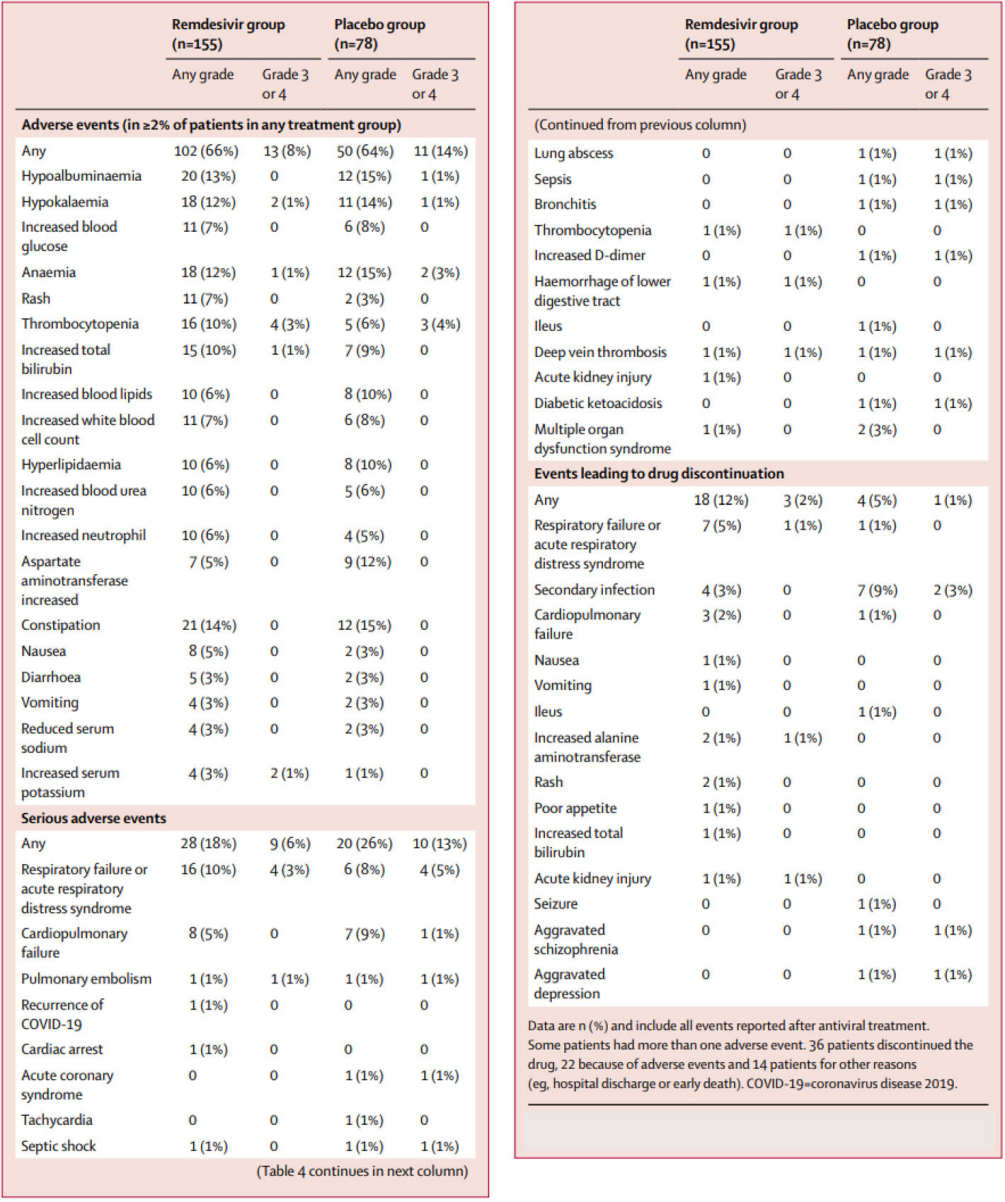
Published AE table for case study 1—randomised controlled trial of remdesivir in adults with severe COVID-19

**Fig. 2:**
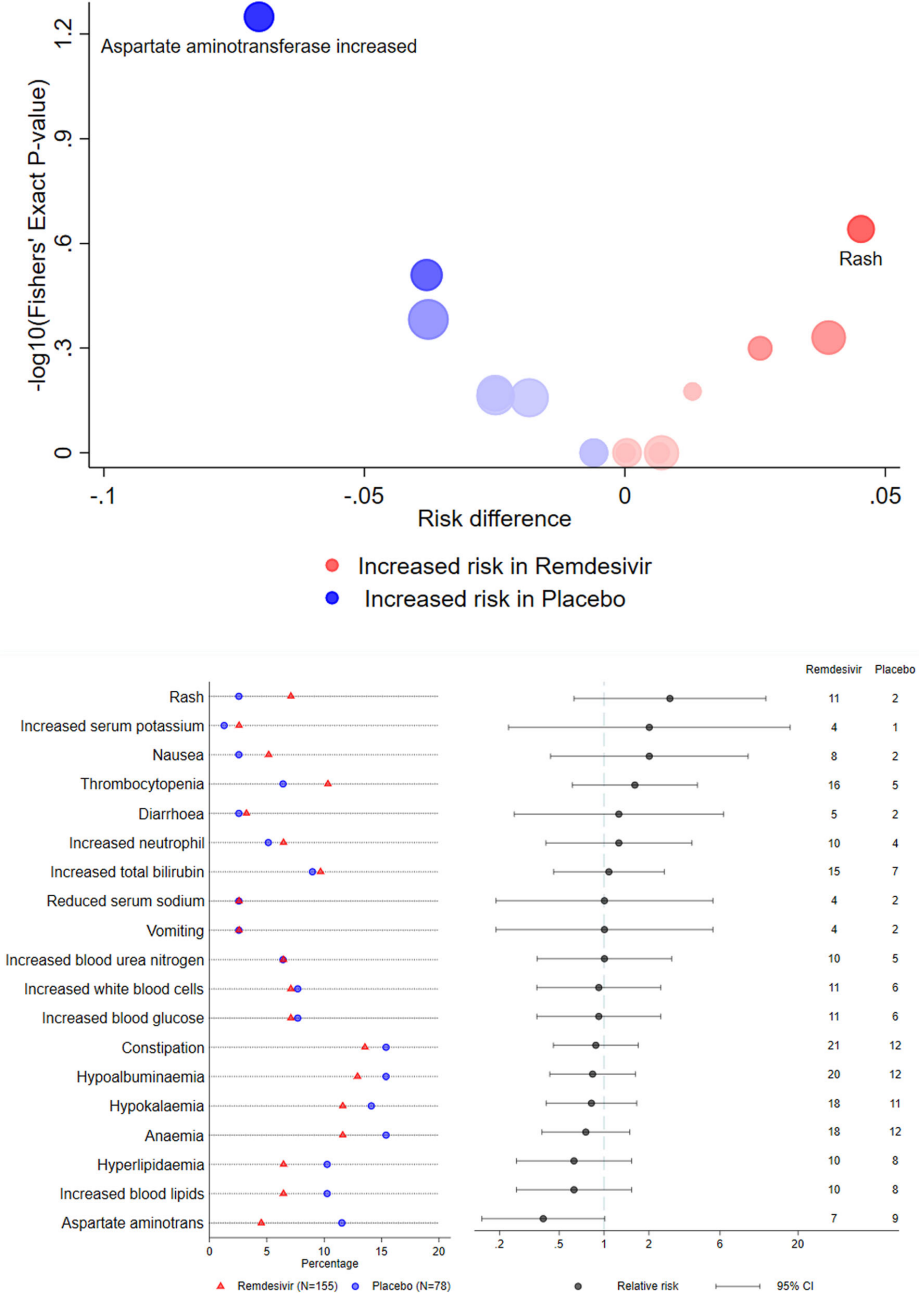
Visual representations of AE data for case study 1—randomised controlled trial of remdesivir in adults with severe COVID-19. **a** Volcano plot for adverse events (in ≥ 2% of patients in any arm) between two treatment arms from Wang et al. [22]. The *x*-axis represents the difference in proportions of participants experiencing each adverse event between the treatment arms (intervention–placebo). The *y*-axis represents the *p* value from Fisher’s exact test on the −log10 scale. The centre of the bubble indicates the coordinates for each adverse event. The size of the bubble is proportional to the total number of events for both treatment arms combined. Colour saturation is used to indicate the strength of the treatment effect with red indicating greater risk in the intervention arm and blue indicating greater risk in the placebo arm. Colour saturation corresponds to the −log10(*p* value) for each event. Labels are added to events where they suggest asymmetry. **b** Dot plot for adverse events (in ≥ 2% of patients in any arm) between two treatment arms. The left side of the figure displays the percentage of participants experiencing an adverse event (labelled on the *y*-axis) in the intervention arm with a red triangle and placebo arm with a blue circle. The right side of the figure displays the relative risk and corresponding 95% confidence interval on the log10 scale

Participants experiencing at least one AE were reported in a total of 66% and 64% of remdesivir and placebo participants respectively. The original AE frequency table reported in the publication can be seen in Fig. 1. Three participants who did not receive treatment in the remdesivir arm and 1 participant who withdrew immediately post-randomisation in the placebo arm were not included in the safety analysis. Authors presented AE data in a frequency table that split the information into three sections, reporting (1) all AEs occurring in ≥ 2% of participants in either treatment arm, (2) all serious adverse events (SAEs), and (3) all AEs leading to study treatment discontinuation. As the three sections in the AE table include overlapping AEs, we demonstrate the use of plots for each section in turn with AEs (in ≥ 2% of participants in any treatment arm) presented in the paper (Fig. 2). Plots for SAEs and AEs leading to study treatment discontinuation are included in supplementary materials (Supp. Figure A1a & b, and Supp. Figure A2a & b). The data and the code to produce the plots in this section can be found in Supp. Tables A1, A2 and A3.

Figure 2a displays a volcano plot of the AEs that occurred in ≥ 2% of participants reported in the table displayed in Fig. 1. The risk difference (RD) is plotted against the log-transformed *p* value. In the absence of any disproportionality in harm between arms, we would expect to see a symmetrical curve, with a random scatter of AEs (not related to the drug) centred around the null value, in this case the RD of zero. Compared to the table, the strength of the volcano plot is its provision of an overarching and immediate representation of the AE profile that allows the user to draw attention to a particular signal of interest, but it does not allow a detailed review of all AEs.

In Fig. 2a, we observe a fairly symmetrical scatter for most AEs, with only two AEs contributing to the asymmetry. These events have been selected to be labelled in the plot to draw attention to them. Labelling shows that the clear outlier is *increased aspartate aminotransferase* which occurs more frequently in the placebo arm (9 (12%) vs 7 (5%)). The greatest harm signal in the remdesivir arm is for *rash* (2 (3%) vs 11 (7%)). *Rash* was not reported by authors in the text, but *increased aspartate aminotransferase* was one of the most common events in the placebo arm. Not reporting rash in the text for the remdesivir arm demonstrates the subjectivity in describing AEs by arm which may make it a less transparent way to communicate results compared to a visual tool. Often common events are mixed up with potentially important differences indicative of ARs.

The same data are presented in Fig. 2b in a dot plot. The dot plot provides a way to simultaneously evaluate both the relative and absolute risk for each AE. In this plot, the relative risk (RR) with accompanying 95% CIs has been plotted on the right hand side and AEs are ordered from the bottom to the top by increasing RR. The 95% CIs can be used both as a means to assess the precision of the risk estimate and the strength of evidence against a null hypothesis of no difference. This can be done through examining the position of the lower or upper CI in comparison to the value of no difference, though we discourage interpretation of CIs fixated only on whether or not they cross the summary statistic value of no difference. This is in line with the CONSORT harms extension that discourages hypothesis-based inference due to a lack of power to test and the increased false discovery rate [11].

In Fig. 2b, we can see that the point estimates of the RRs are evenly distributed on either side of the vertical line of no difference (RR = 1) and that there is great uncertainty in these estimates. The point estimate furthest away from one communicates an increased risk of *increased aspartate aminotransferase* in the placebo arm, and the upper 95% CI excludes the value of no difference. The corresponding absolute risks can be read from the left hand side of the graph with the number of AEs by arm included in the right hand side of the plot. This detailed information on absolute values can be especially useful in situations such as when the event rate is low. In contrast to the volcano plot, the dot plot provides information that requires more assimilation but contains more data.

The volcano and dot plots for SAEs reported in Fig. 1 can be seen in Supp. Figure A1a and b. The data and the code to produce the plots can be found in Supp. Tables A4 and A5. In the original article, authors’ only description of SAEs was for the total number ‘28 (18%) serious adverse events were reported in the remdesivir arm and 20 (26%) were reported in the control arm.’ What is notable in Supp. Fig A1a is that due to the lower event rates, the volcano plot of the SAEs is harder to interpret as there are a number of overlapping events that have the same RD and *p* value and therefore cannot be uniquely seen. The volcano plot does display a highly asymmetrical pattern and highlights a signal for a higher risk in the placebo arm of *cardiopulmonary failure* (7 (9%) vs 8 (5%)) and *multiple organ dysfunction syndrome* (2 (3%) vs 1(1%)). The prominence in the volcano plot of *multiple organ dysfunction syndrome* is arguably overemphasised in the plot considering the extremely low event rate. This provides an example where the use of the volcano plot alone to draw inference in the absence of other information could provide a misleading impression.

The dot plot (Supp. Figure A1b) provides a more accurate display of the SAE terms compared to the corresponding volcano plot. It can be seen that the point estimates are generally more in favour of remdesivir than placebo, as 13 RR point estimates sit to the left of the null value compared to 6 with placebo. However, there is great uncertainty in the risk estimates with all of them generously overlapping the value of no difference. On the left hand side of the dot plot, it can be seen that the absolute risk for each SAE is low with many events containing zero events in one of the arms.

The authors reported ‘Remdesivir was stopped early because of adverse events in 18 (12%) patients versus four (5%) patients who stopped placebo early.’ The volcano plot for discontinuations due to AEs in Supp. Figure A2a suffers the same limitation for interpretations as the previous example with SAEs due to the low event rate.

The volcano plot highlights a signal for harm in the placebo arm for serious *secondary infections* (7 (9%) vs 4 (3%)), which is not mentioned in the authors’ summary, and while *respiratory failure* is notable in the plot, the strength of evidence is less than for serious *secondary infections*. However, it should be noted that the plot can only display the numerical difference and does not convey whether secondary infections are less of a concern than respiratory failure and therefore a smaller difference in respiratory failure may be of more importance than a greater one in secondary infections. The dot plot (Supp. Fig A2b) supported the same message as the volcano plot for *secondary infections*, but the higher rate for *respiratory failure* in the remdesivir arm is more notable in this plot. There is greater uncertainty in this estimate which is displayed through the 95% CI, and it can be seen that the count is low (1 (1%) vs 7 (5%)).

Finally, the authors provided a descriptive summary of AEs in the text. The authors selected to list the names of the ‘most common’ events by arm. The definition of ‘most common’ was not provided. They reported the six most common AEs in the remdesivir arm and the seven most common AEs in the placebo arm. The usefulness of highlighting the most common events, listing them by arm, is unclear as they may not be clinically important or numerically imbalanced between arms. In this instance, five of those listed were the same in both arms, and without any further qualification, interpretation is difficult. Picking out the most commonly occurring AEs by arm means the AEs could just relate to the underlying infection or participant comorbidity when they are the same across both arms. If they are different between arms, it could indicate a drug effect.

### Case study 2: Randomised controlled trial of intermittent intraputamenal glial cell line-derived neurotrophic factor in Parkinson’s disease

#### Study design

Whone et al. performed a single-centre, randomised, double-blind, placebo-controlled trial of glial cell line-derived neurotrophic factor (GDNF) for patients with Parkinson’s disease delivered using intermittent intraputamenal convection-enhanced delivery via a skull-mounted transcutaneous port [23]. GDNF has shown mixed results in Parkinson’s patients to date which the study authors attribute to a potential insufficient GDNF exposure across the putamen. The primary efficacy outcome was the percentage change from baseline in the OFF state of the Unified Parkinson’s Disease Rating Scale (UPDRS) motor score (part III) after 40 weeks of treatment. The trial was powered to detect a difference of 20 points in percentage change, assuming a standard deviation of 20%, with 80% power and two-sided type one error of 5%. Six participants were randomised (2:1) into a pilot phase, and 35 participants were randomised (1:1) into the primary phase to receive either infusions of GDNF or placebo every 4 weeks for 40 weeks.

All participants regardless of treatment arm assignment had to undergo robot-assisted surgery for implantation of a skull-mounted port to allow administration of the drug. This necessary surgery to deliver the active drug or placebo added an additional layer of complexity to the reporting of AEs in the trial.

AEs, routine laboratory tests, and anti-GDNF serum antibodies were used to assess safety. Treatment-emergent adverse events (TEAEs) were defined as any event starting on or after the date of the first dose of study medication, and analysis included participants from both the pilot and primary phase. *Dyskinesia*, *falls*, *adverse changes in mood*, and *impulsivity* were pre-specified as AEs of special interest.

#### Results

**Fig. 3:**
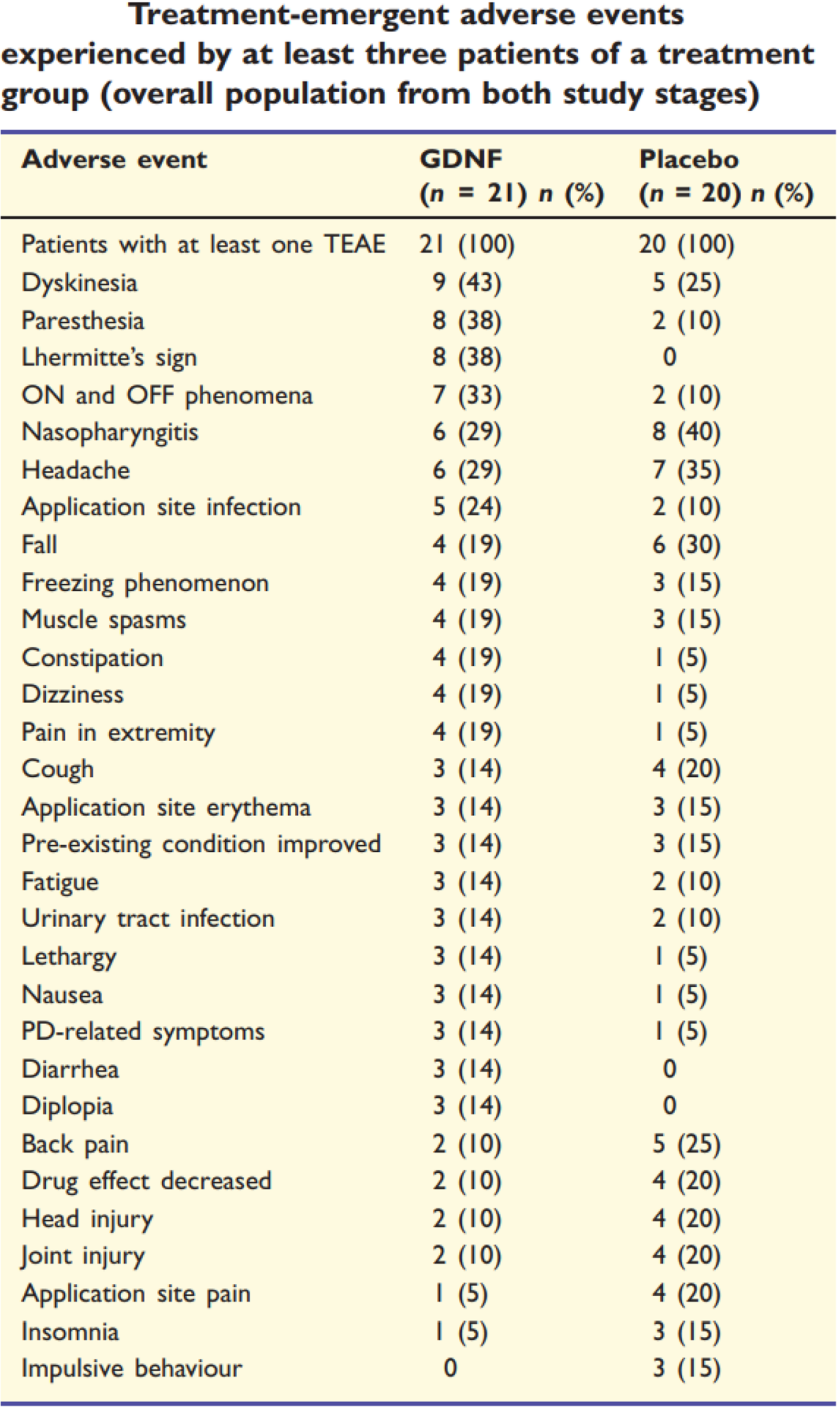
Published AE table for case study 2—randomised controlled trial of GDNF in Parkinson’s disease

In the publication, the authors selected to report all treatment-emergent adverse events (TEAEs) experienced by at least three participants in either treatment arm (shown in Fig. 3).

In the abstract, the authors reported that ‘GDNF appeared to be well tolerated and safe, and no drug-related serious adverse events were reported.’ This message was reiterated in the discussion. On inspection of the AE table in Fig. 3, it can be seen that there is a signal for imbalance in *paraesthesia* (GDNF 8 (38%), placebo 2 (10%)), *Lhermitte’s sign* (GDNF 8 (38%), placebo 0 (0%)), and *ON/OFF phenomena* (GDNF 7 (33%), placebo 2 (10%)).

**Fig. 4:**
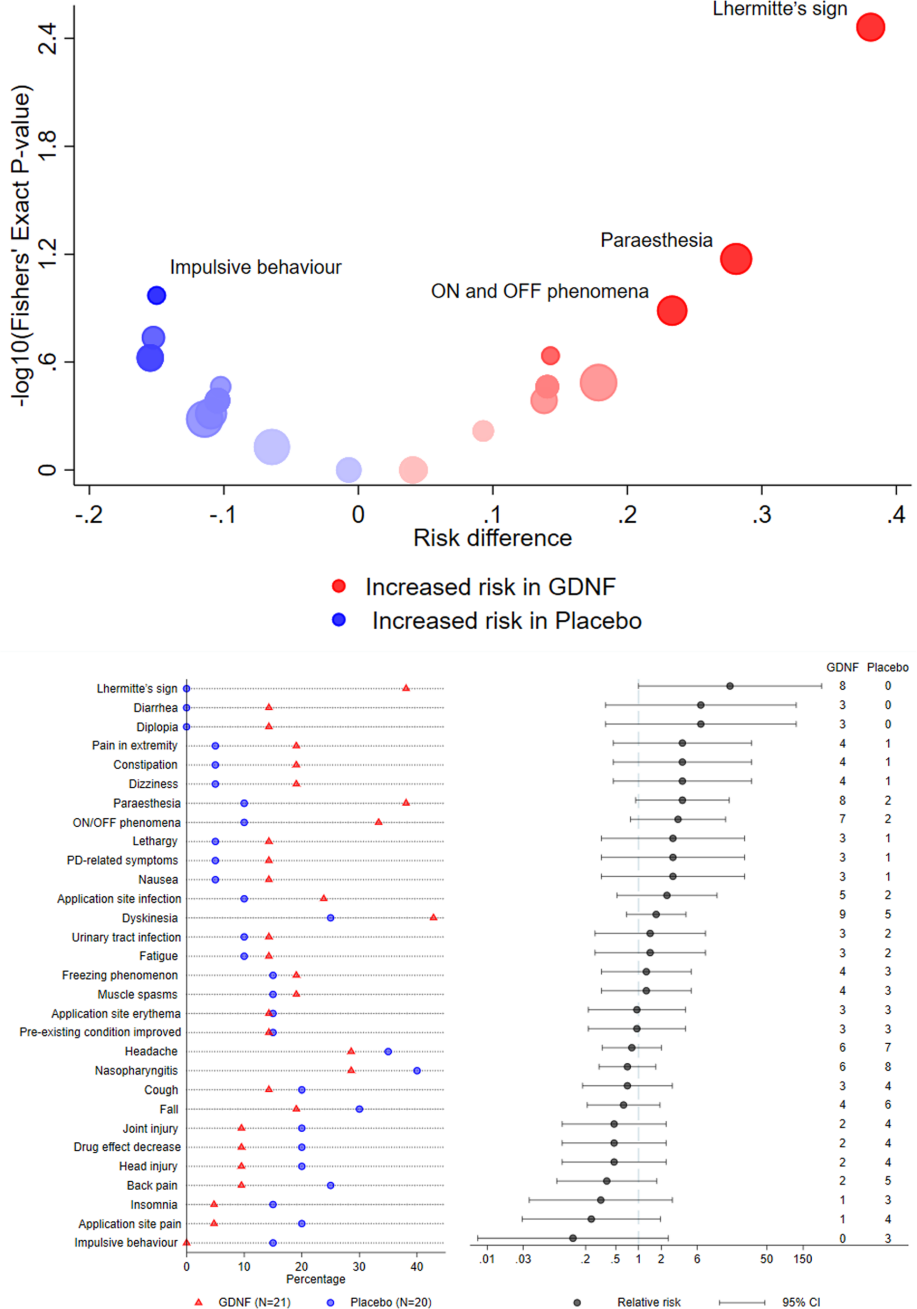
Visual representations of AE data for case study 2—randomised controlled trial of GDNF in Parkinson’s disease. **a** Volcano plot for adverse events experienced by at least three participants in either treatment arm from Whone et al. The *x*-axis represents the difference in proportions of participants experiencing each adverse event between the treatment arms (intervention–placebo). The *y*-axis represents the *p* value from Fisher’s exact test on the −log10 scale. The centre of the bubble indicates the coordinates for each adverse event. The size of the bubble is proportional to the total number of events for both treatment arms combined. Colour saturation is used to indicate the strength of the treatment effect with red indicating greater risk in the intervention arm and blue indicating greater risk in the placebo arm. Colour saturation corresponds to the −log10(*p* value) for each event. Labels are added to events where they suggest asymmetry. **b** Dot plot for adverse events experienced by at least three participants in either treatment arm from Whone et al. The left side of the figure displays the percentage of participants experiencing an adverse event (labelled on the *y*-axis) in the intervention arm with a red triangle and placebo arm with a blue circle. The right side of the figure displays the relative risk and corresponding 95% confidence interval on the log10 scale

Figure 4a displays the information from the published AE table in a volcano plot. The data and the code to produce the plots in this section be found in Supp. Tables A8, A9 and A10. In contrast to the information presented in the authors’ table (Fig. 3), the greatest imbalance of events for *Lhermitte’s sign* is immediately communicated via the volcano plot, and it can be seen that the risk is greater in the GDNF arm compared to placebo. There are also three other events of note: two in the GDNF arm (*paraesthesia* and *ON/OFF phenomena*) and *impulsive behaviour* in the placebo arm. We selected these to be labelled in the plot as removing these events would leave a symmetrical curve. These events are less apparent from a review of the article’s frequency table. The authors did comment on *paraesthesia*, *Lhermitte’s sign*, and *ON/OFF phenomena* within the text, but they did not report on the increased risk of *impulsive behaviour* due to the rules used to select events to report.

The same data is presented in Fig. 4b in a dot plot. While the volcano plot brings high focus attention to the most extreme events, the dot plot provides a more detailed summary of all events, with both an absolute risk and relative risk presented. In this example, we can see that the red triangles, which depict the absolute risk of events in the GDNF arm, indicate a greater prevalence of events in the GDNF arm. We also see there are several events where the RR lower 95% CI bound almost completely lies above one (not quite excluding the value of no difference) suggesting an increased risk of *Lhermitte’s sign*, *paraesthesia*, and *ON/OFF phenomena* in the GDNF arm.

We can see that summarising the same AE data in a graphical display allows for both the full picture and a comprehendible summary, helping communicate this information. The author’s use of ad hoc rule-based thresholds (e.g. ≥ 3), either as a minimum number of events for inclusion in the table or as a between-arm difference to highlight a signal, can result in important AE information being omitted and not communicated.

As well as data in the table, the authors included a commendable comprehensive descriptive summary of these in the text. The authors clarified that no participants had a TEAE that led to discontinuation of the study medication. The authors listed out some of the TEAEs where there was a between-arm difference of ≥ 3 participants experiencing an event in the GDNF arm compared to the placebo arm. It was not clear why the threshold of a difference in ≥ 3 participants in the GDNF arm was used, why they selected only some AEs to highlight in the text, and why they did not highlight TEAEs that occurred more frequently in the placebo arm. It is possible authors selected a subset they subjectively assessed to be most relevant that met their original ad hoc rules as a means to provide a comprehendible summary to readers.

In the publication text, the authors also reported a balance of the overall frequencies for the pre-specified AEs of special interest in each treatment arm (GDNF 13 (62%), placebo 11 (55%)). Event-specific information is included in Fig. 3 for three of the four pre-specified events, but no information is provided on *adverse changes in mood*; this may be because no events were reported, but it is not clear. In the publication, text information on serious AEs by treatment arm (GDNF 5 (24%), placebo 0 (0%)) is reported, with authors noting that none of these events was related to study medication.

The authors also reported on device-related adverse events being dominated by *port site infections* and *local hypertrophic scarring*, noting that many of these events occurred post-surgery but pre-treatment. These post-surgery, pre-treatment events do not meet the definition of TEAEs, and therefore, a summary of AEs associated to surgery and the device was omitted from the article. This information may have been helpful for real-world application as patients will require that a port is fitted in order to receive the drug.

An additional example is provided in the supplementary material (tables A11, A12 and A13 and figure A3a and b) to demonstrate implementation of the Stata aevolcano and aedot commands when individual participant data is available.

## Discussion

There have been many different graphical displays proposed [10, 13, 15], for AEs and the value and appropriateness of these will depend on the variable type, the number of AEs, and context for display. In this article, we have demonstrated two methods suitable for use in the final analysis of multiple binary outcomes and contrasted these with the use of frequency tables to display the same information.

We have used data from randomised trials in COVID-19 [22] and Parkinson’s disease to demonstrate how these visual displays can provide an alternative way to communicate risks of harm compared to traditional frequency tables. We found that assessment of AE data by review of tabulations, volcano plot, and dot plot each provided a different emphasis. In this article, we proposed inferences based on the reviews of these plots which differ in emphasis from the authors’ published descriptions of their frequency tables.

Contemporary trial statisticians require statistical knowledge across many different domains to undertake the best statistical practice. As the field is advancing in each domain year-on-year, it can be challenging for applied statisticians to learn and programme new methods at the rate of emerging progress. Supporting applied statisticians with accessible software tools can enable them to undertake the best practice and enable early adoption of emerging statistical methods. We have demonstrated and provided accessible software code to produce graphical tools that would otherwise require lengthy complex coding with the aim to accelerate the translation of better AE analysis methods into practice.

The volcano plot had the benefit in immediate communication of extreme differences in AE rates through fast and simple evaluation of asymmetry in the plot. It provided a means to assess the ‘profile’ of AEs but without the detail, suggesting the approach as a highly effective way to communicate signals for ARs. Users are able to highlight AEs of concern by adding labels to the required events in the plot. The volcano plot was found to be less effective when there were either few AEs in total, or where several AEs shared the same frequency, which occurs most often when the event rates are low. As a result, the volcano plot will be most useful when there are around 10 events or more and are also not dominated by low frequency counts, e.g. 0 and 1. The advantage of the volcano plot is also what brings its disadvantage, i.e. its effective ability to communicate a signal for an AR does not transparently highlight the precision of the estimate, and we found it was possible to give a misleading impression in some scenarios when the event numbers were small. Researchers need to be cautious when constructing and interpreting a volcano plot and check if they assess the communication to be a fair representation of the raw data and message delivered.

The dot plot provided a very different way to communicate results compared to the volcano plot and allows more detailed information to be included. The dot plot required more involved appraisal, but arguably shorter than that required when reviewing a frequency table. The dot plot could be viewed as providing a substitute to an AE frequency table, whereas the use of a volcano plot will likely still require presentation of more detailed information in a table. While the dot plot laudably contains both the absolute risk by arm and relative between-arm comparison, we found the inclusion of 95% CIs for the relative statistic strongly encourages the temptation to interpret the 95% CI as a test of no statistical difference—which in turn may encourage false flagging as well as missed signals.

The volcano plot encourages review of the risk profile as a whole, as events are not all labelled, whereas the dot plot allows for more scrutiny by clinical investigators who may want to mentally group similar body system terms in their interpretation, e.g. noting the direction and strength of effect across multiple cardiovascular terms rather than viewing each as individual events.

This study was limited to the data selected for inclusion in the original articles and has only demonstrated use in two example trials. Trials of differing sample size, event rates, and AE complexity may highlight further advantages and disadvantages for visual displays.

## Conclusion

Visual displays of AE data provide an effective way to communicate harm and support different perspectives on interpretation and aid communication. Visualisations can aid investigators to assimilate large volumes of data and also encourage informal between-arm comparisons in contrast to tables. Care in construction of visual displays is needed as they can overemphasise treatment effects in some circumstances. The use of tables, dot plots, and volcano plots can encourage differing interpretations. Volcano plots bring high focus attention to the most extreme events, and the dot plot provides a more detailed summary of all events. We recommend trialists examine both crude numbers along with graphical display to help draw inferences.

## Supplementary Information

**Additional file 1.** Supplementary file

## Data Availability

All data used in this study are included in this published article and its supplementary files. The data is also publically available in the original case study publications.

## Abbreviations

AE: Adverse event
AR: Adverse reaction
CI: Confidence interval
CONSORT: Consolidated Standards of Reporting Trials
COVID-19: Coronavirus disease 2019
FDR: False discovery rate
GDNF: Glial cell line-derived neurotrophic factor
MERS: Middle East respiratory syndrome
RCT: Randomised controlled trial
RD: Risk difference
RR: Relative risk
SAE: Serious adverse event
TEAE: Treatment-emergent adverse event
UPDRS: Unified Parkinson’s disease rating scale
