# Supplementary material for: Advantages of visualisations to evaluate and communicate adverse event information in randomised controlled trials
Source: Trials. 2020 Dec 22;21:1028. doi: 10.1186/s13063-020-04903-0 (PMC7754702; doi:10.1186/s13063-020-04903-0)
Supplement: Supplementary file 1 — Additional file 1. Supplementary file [file 13063_2020_4903_MOESM1_ESM.docx]

**Appendix**

**Case study 1:** **Remdesivir in adults with severe COVID-19: a randomised, double-blind, placebo-controlled, multicentre trial**

Table A1: Stata dataset used to produce figure 1 in main article. Data taken from table 1 of main article for adverse events occurring in *≥ 2% of participants in either treatment group*

| adverse_events | Remdesivir_AG_n | Remdesivir_G34_n | Remdesivir_N | Placebo_AG_n | Placebo_G34_n | Placebo_N |
| --- | --- | --- | --- | --- | --- | --- |
| Any | 102 | 13 | 155 | 50 | 11 | 78 |
| Hypoalbuminaemia | 20 | 0 | 155 | 12 | 1 | 78 |
| Hypokalaemia | 18 | 2 | 155 | 11 | 1 | 78 |
| Increased blood glucose | 11 | 0 | 155 | 6 | 0 | 78 |
| Anaemia | 18 | 1 | 155 | 12 | 2 | 78 |
| Rash | 11 | 0 | 155 | 2 | 0 | 78 |
| Thrombocytopenia | 16 | 4 | 155 | 5 | 3 | 78 |
| Increased total bilirubin | 15 | 1 | 155 | 7 | 0 | 78 |
| Increased blood lipids | 10 | 0 | 155 | 8 | 0 | 78 |
| Increased white blood cell count | 11 | 0 | 155 | 6 | 0 | 78 |
| Hyperlipidaemia | 10 | 0 | 155 | 8 | 0 | 78 |
| Increased blood urea nitrogen | 10 | 0 | 155 | 5 | 0 | 78 |
| Increased neutrophil | 10 | 0 | 155 | 4 | 0 | 78 |
| Aspartate aminotransferase increased | 7 | 0 | 155 | 9 | 0 | 78 |
| Constipation | 21 | 0 | 155 | 12 | 0 | 78 |
| Nausea | 8 | 0 | 155 | 2 | 0 | 78 |
| Diarrhoea | 5 | 0 | 155 | 2 | 0 | 78 |
| Vomiting | 4 | 0 | 155 | 2 | 0 | 78 |
| Reduced serum sodium | 4 | 0 | 155 | 2 | 0 | 78 |
| Increased serum potassium | 4 | 2 | 155 | 1 | 0 | 78 |

Table A2: Variable description for dataset displayed in table A1

| **Variable name** | **Variable description** |
| --- | --- |
| adverse_event | Adverse event name |
| Remdesivir_AG_n | number of events in Remdesivir arm |
| Remdesivir_G34_n | number of grade 3 and 4 events in Remdesivir arm |
| Remdesivir_N | total number of participants in Remdesivir arm |
| Placebo_AG_n | number of events in placebo arm |
| Placebo_G34_n | number of grade 3 and 4 events in placebo arm |
| Placebo_N | total number of participants in placebo arm |

Table A3: Code required to produce the volcano and dot plot in figure 1 using data from table A1

| **Stata code** | **Description** |
| --- | --- |
| - use dataset_name.dta, clear | Call dataset name saved data from table A1 |
| - drop if adverse_event=="Any" | Drop the observation that summarises overall number of AEs |
| - aevolcs adverse_event, n1( Remdesivir_AG_n ) n2( Placebo_AG_n ) tot1( Remdesivir_N ) tot2( Placebo_N ) legendyn(1) legend1(Increased risk in Remdesivir) legend2(Increased risk in Placebo) labelyn(1) labang(0) labpos(6) labgap(2) label(0.55) | Command to produce volcano plot. Options n1, n2, tot1 and tot1 required. Other options used to format figure. |
| - aedots adverseevents , n1( Remdesivir_AG_n ) n2( Placebo_AG_n ) tot1( Remdesivir_N ) tot2( Placebo_N ) logoff(1) leftcolor1(red) leftcolor2(blue) leftsymb1(triangle) legendleft1(Remdesivir (N=155)) legendleft2(Placebo (N=78)) brightmargin(3.5) leftlabsize(1.3) rightxline(1) rightxlabel(0.2 0.5 1 2 6 20) nummargin(1) margin(30) event1pos(35) event2pos(90) event1name(Remdesivir) event2name(Placebo) | Command to produce dot plot. Options n1, n2, tot1 and tot1 required. Other options used to format figure. |

Table A4: Stata dataset used to produce figure A1. Data taken from table 1 of main article for *serious adverse events*

| adverse_events | Remdesivir_AG_n | Remdesivir_G34_n | Remdesivir_N | Placebo_AG_n | Placebo_G34_n | Placebo_N |
| --- | --- | --- | --- | --- | --- | --- |
| Any | 28 | 9 | 155 | 20 | 10 | 78 |
| Respiratory failure or acute respiratory distress syndrome | 16 | 4 | 155 | 6 | 4 | 78 |
| Cardiopulmonary failure | 8 | 0 | 155 | 7 | 1 | 78 |
| Pulmonary embolism | 1 | 1 | 155 | 1 | 1 | 78 |
| Recurrence of COVID-19 | 1 | 0 | 155 | 0 | 0 | 78 |
| Cardiac arrest | 1 | 0 | 155 | 0 | 0 | 78 |
| Acute coronary syndrome | 0 | 0 | 155 | 1 | 1 | 78 |
| Tachycardia | 0 | 0 | 155 | 1 | 0 | 78 |
| Septic shock | 1 | 0 | 155 | 1 | 1 | 78 |
| Lung abscess | 0 | 0 | 155 | 1 | 1 | 78 |
| Sepsis | 0 | 0 | 155 | 1 | 1 | 78 |
| Bronchitis | 0 | 0 | 155 | 1 | 1 | 78 |
| Thrombocytopenia | 1 | 1 | 155 | 0 | 0 | 78 |
| Increased D-dimer | 0 | 0 | 155 | 1 | 1 | 78 |
| Haemorrhage of lower digestive tract | 1 | 1 | 155 | 0 | 0 | 78 |
| Ileus | 0 | 0 | 155 | 1 | 0 | 78 |
| Deep vein thrombosis | 1 | 1 | 155 | 1 | 1 | 78 |
| Acute kidney injury | 1 | 0 | 155 | 0 | 0 | 78 |
| Diabetic ketoacidosis | 0 | 0 | 155 | 1 | 1 | 78 |
| Multiple organ dysfunction syndrome | 1 | 0 | 155 | 2 | 0 | 78 |

Note: Variable description as per table A2

Table A5: Code required to produce the volcano and dot plot in figure A1 using data from table A4

| **Stata code** | **Description** |
| --- | --- |
| - use dataset_name.dta, clear | Call dataset name saved data from table A4 |
| - drop if adverse_event=="Any" | Drop the observation that summarises overall number of AEs |
| - aevolcs adverse_events , n1( Remdesivir_AG_n ) n2( Placebo_AG_n ) tot1( Remdesivir_N ) tot2( Placebo_N ) labelyn(1) label(0.5) labang(0) labpos(4) labgap(3) legendyn(1) legend1(Increased risk in Remdesivir) legend2(Increased risk in Placebo) | Command to produce volcano plot. Options n1, n2, tot1 and tot1 required. Other options used to format figure. |
| - aedots adverseevents , n1( Remdesivir_AG_n ) n2( Placebo_AG_n ) tot1( Remdesivir_N ) tot2( Placebo_N ) leftcolor1(red) leftcolor2(blue) leftsymb1(triangle) legendleft1(Remdesivir (N=155)) legendleft2(Placebo (N=78)) brightmargin(3.5) leftlabsize(1.3) logoff(1) rightxlabel(0.01 0.03 0.2 0.5 1 2 6 50 ) nummargin(1) margin(30) event1pos(90) event2pos(500) event1name(Remdesivir) event2name(Placebo) | Command to produce dot plot. Options n1, n2, tot1 and tot1 required. Other options used to format figure. |

**
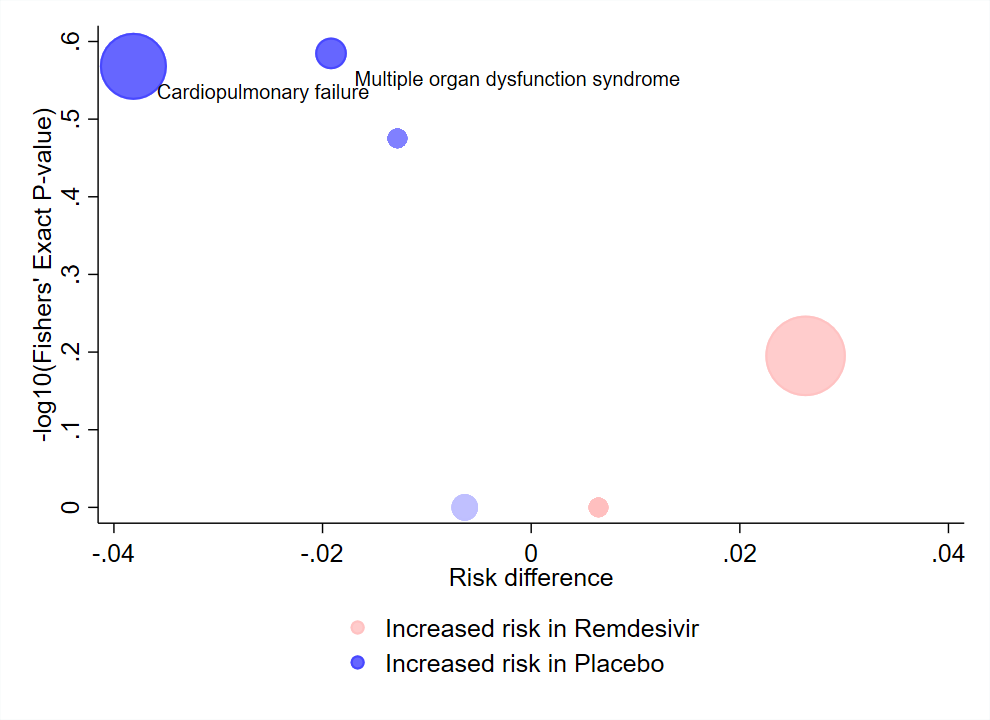
** **
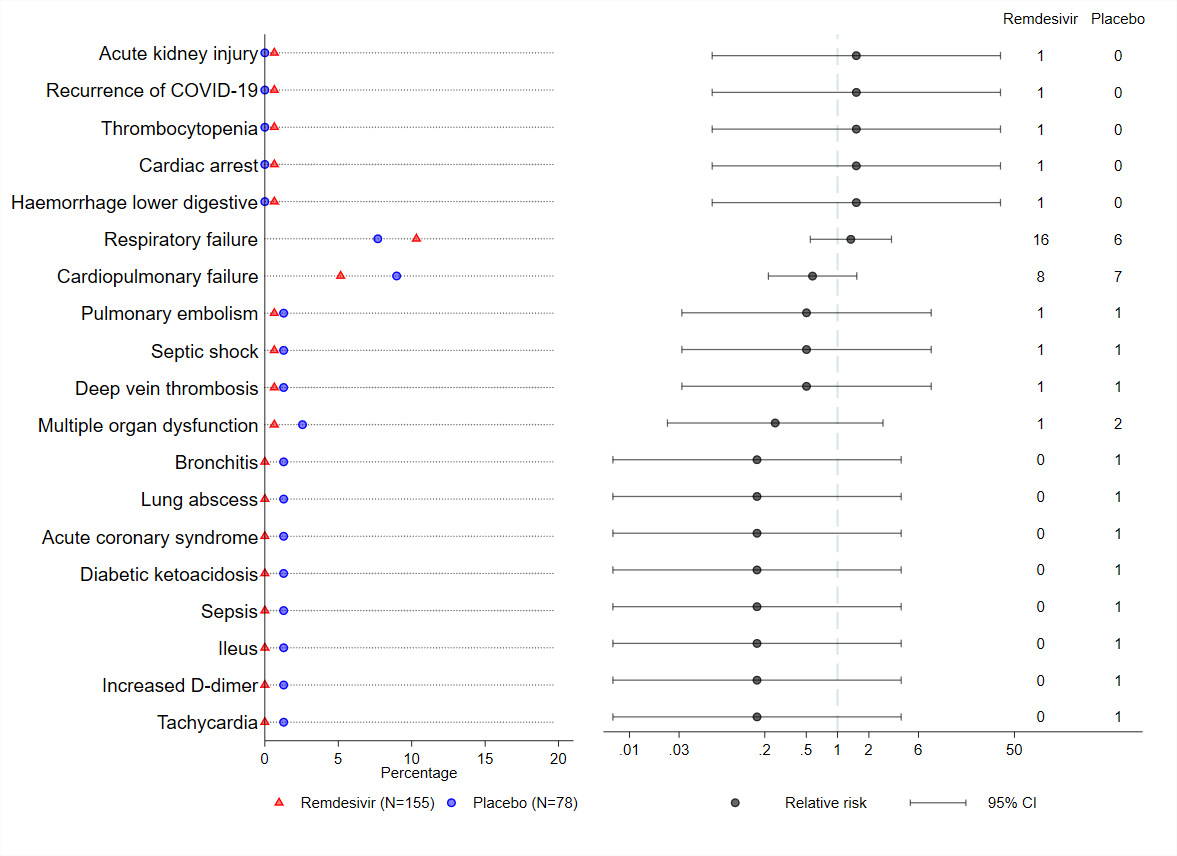
**

**Figure A1a:** Volcano Plot for **serious adverse events** between two treatment arms from Wang et al 2020. *The x-axis represents the difference in proportions of participants experiencing each adverse event between the treatment arms (intervention – placebo). The y-axis represents the p-value from a Fisher’s exact test on the -log10 scale. The centre of the bubble indicates the coordinates for each adverse event. The size of the bubble is proportional to the total number of events for both treatment groups combined. Colour is used to indicate direction of treatment effect with red indicating greater risk in the intervention group and blue indicating greater risk in the placebo group. Colour saturation corresponds to the -log10(p-value) for each event.*

**Figure A1b:** Dot Plot for **serious adverse events** between two treatment arms. *The left side of the figure displays the percentage of participants experiencing an adverse event (labelled on the y-axis) in the intervention group with a red triangle and placebo group with a blue circle. The right side of the figure displays the relative risk and corresponding 95% confidence interval on the log10 scale.*

Table A6: Stata dataset used to produce figure A2. Data taken from table 1 of main article for events *leading to study treatment discontinuation*

| adverse_events | Remdesivir_AG_n | Remdesivir_G34_n | Remdesivir_N | Placebo_AG_n | Placebo_G34_n | Placebo_N |
| --- | --- | --- | --- | --- | --- | --- |
| Any | 18 | 3 | 155 | 4 | 1 | 78 |
| Respiratory failure or acute respiratory distress syndrome | 7 | 1 | 155 | 1 | 0 | 78 |
| Secondary infection | 4 | 0 | 155 | 7 | 2 | 78 |
| Cardiopulmonary failure | 3 | 0 | 155 | 1 | 0 | 78 |
| Nausea | 1 | 0 | 155 | 0 | 0 | 78 |
| Vomiting | 1 | 0 | 155 | 0 | 0 | 78 |
| Ileus | 0 | 0 | 155 | 1 | 0 | 78 |
| Increased alanine aminotransferase | 2 | 1 | 155 | 0 | 0 | 78 |
| Rash | 2 | 0 | 155 | 0 | 0 | 78 |
| Poor appetite | 1 | 0 | 155 | 0 | 0 | 78 |
| Increased total bilirubin | 1 | 0 | 155 | 0 | 0 | 78 |
| Acute kidney injury | 1 | 1 | 155 | 0 | 0 | 78 |
| Seizure | 0 | 0 | 155 | 1 | 0 | 78 |
| Aggravated schizophrenia | 0 | 0 | 155 | 1 | 1 | 78 |
| Aggravated depression | 0 | 0 | 155 | 1 | 1 | 78 |

Note: Variable description as per table A2

Table A7: Code required to produce the volcano and dot plot in figure A2 using data from table A6

| **Stata code** | **Description** |
| --- | --- |
| - use dataset_name.dta, clear | Call dataset name saved data from table A6 |
| - drop if adverse_event=="Any" | Drop the observation that summarises overall number of AEs |
| - aevolcs adverse_events , n1( Remdesivir_AG_n ) n2( Placebo_AG_n ) tot1( Remdesivir_N ) tot2( Placebo_N ) yaxismax(0.3) labelyn(1) label(0.5) labpos(12) labang(0) labgap(5) xaxismin(-0.02) xaxismax(0.02) xaxisdp(0.02) xaxisticks(5) legendyn(1) legend1(Increased risk in Remdesivir) legend2(Increased risk in Placebo) | Command to produce volcano plot. Options n1, n2, tot1 and tot1 required. Other options used to format figure. |
| - aedots Aadverseevents , n1( Remdesivir_AG_n ) n2( Placebo_AG_n ) tot1( Remdesivir_N ) tot2( Placebo_N ) leftcolor1(red) leftcolor2(blue) leftsymb1(triangle) legendleft1(Remdesivir (N=155)) legendleft2(Placebo (N=78)) brightmargin(5) leftlabsize(1.3) nummargin(1) margin(30) event1pos(110) event2pos(600) event1name(Remdesivir) event2name(Placebo) logoff(1) rightxlabel(0.01 0.03 0.2 0.5 1 2 6 50 ) | Command to produce dot plot. Options n1, n2, tot1 and tot1 required. Other options used to format figure. |


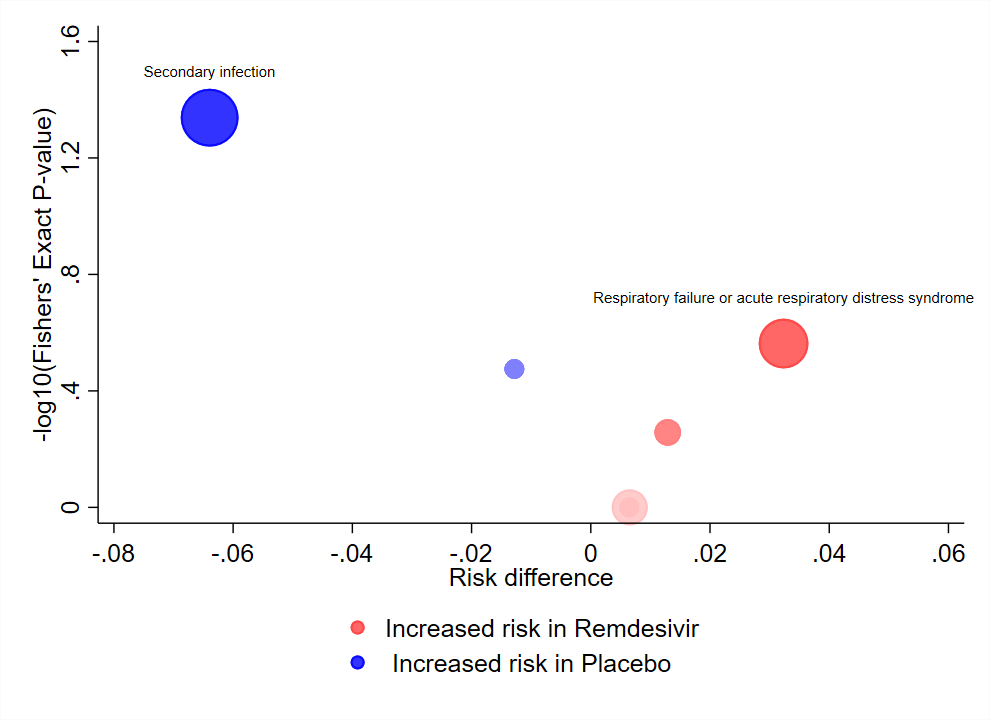


**
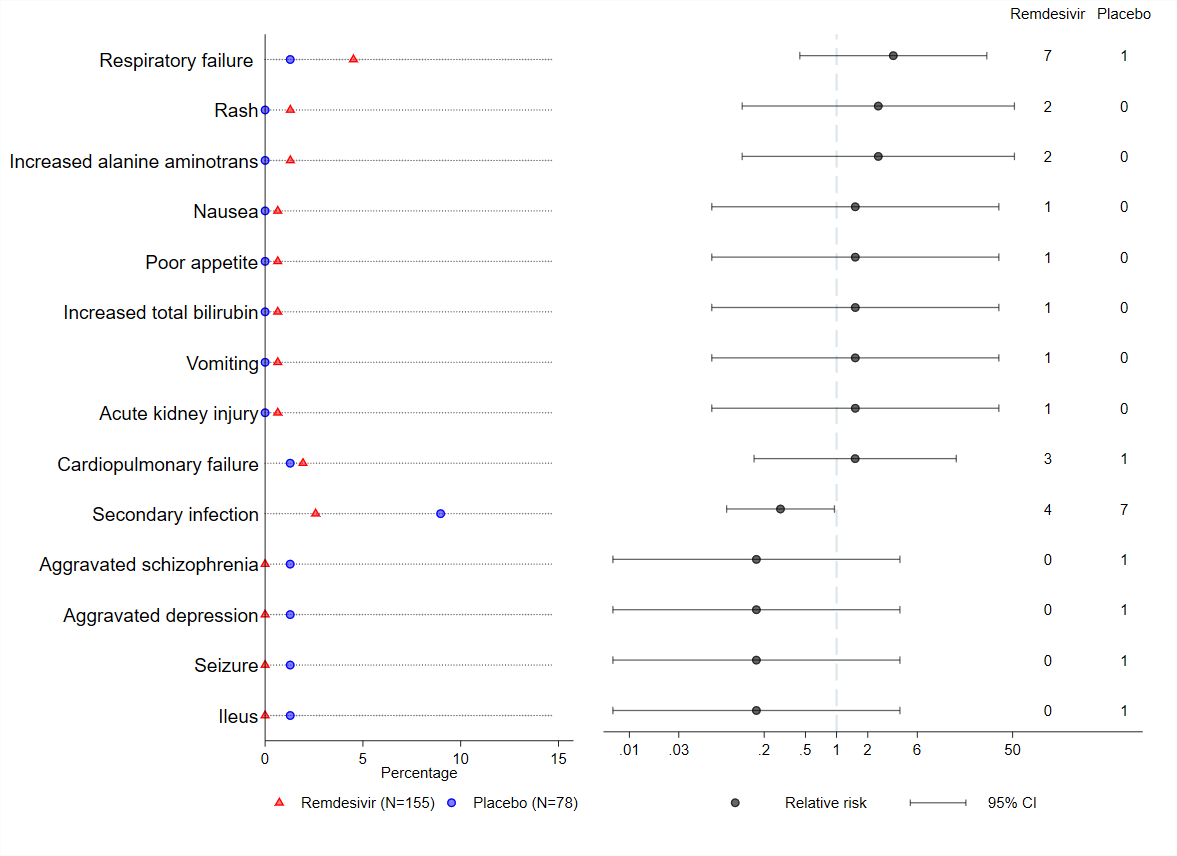
**

**Figure A2a:** Volcano Plot for **adverse events leading to discontinuation** between two treatment arms from Wang et al 2020. *The x-axis represents the difference in proportions of participants experiencing each adverse event between the treatment arms (intervention – placebo). The y-axis represents the p-value from a Fisher’s exact test on the -log10 scale. The centre of the bubble indicates the coordinates for each adverse event. The size of the bubble is proportional to the total number of events for both treatment groups combined. Colour is used to indicate direction of treatment effect with red indicating greater risk in the intervention group and blue indicating greater risk in the placebo group. Colour saturation corresponds to the -log10(p-value) for each event.*

**Figure A2b:** Dot Plot for **adverse events leading to discontinuation** between two treatment arms. *The left side of the figure displays the percentage of participants experiencing an adverse event (labelled on the y-axis) in the intervention group with a red triangle and placebo group with a blue circle. The right side of the figure displays the relative risk and corresponding 95% confidence interval on the log10 scale.*

**Case study 2: Randomized trial of intermittent intraputamenal glial cell line-derived neurotrophic factor in Parkinson’s disease**

Table A8: Stata dataset used to produce figure 2 in main article. Data taken from table 2 of main article for adverse events *experienced by at least three participants in either treatment group*

| adverse_events | gdnf_n | gdnf_N | placebo_n | placebo_N |
| --- | --- | --- | --- | --- |
| Patients with at least one TEAE | 21 | 21 | 20 | 20 |
| Dyskinesia | 9 | 21 | 5 | 20 |
| Paraesthesia | 8 | 21 | 2 | 20 |
| Lhermitte’s sign | 8 | 21 | 0 | 20 |
| ON and OFF phenomena | 7 | 21 | 2 | 20 |
| Nasopharyngitis | 6 | 21 | 8 | 20 |
| Headache | 6 | 21 | 7 | 20 |
| Application site infection | 5 | 21 | 2 | 20 |
| Fall | 4 | 21 | 6 | 20 |
| Freezing phenomenon | 4 | 21 | 3 | 20 |
| Muscle spasms | 4 | 21 | 3 | 20 |
| Constipation | 4 | 21 | 1 | 20 |
| Dizziness | 4 | 21 | 1 | 20 |
| Pain in extremity | 4 | 21 | 1 | 20 |
| Cough | 3 | 21 | 4 | 20 |
| Application site erythema | 3 | 21 | 3 | 20 |
| Pre-existing condition improved | 3 | 21 | 3 | 20 |
| Fatigue | 3 | 21 | 2 | 20 |
| Urinary tract infection | 3 | 21 | 2 | 20 |
| Lethargy | 3 | 21 | 1 | 20 |
| Nausea | 3 | 21 | 1 | 20 |
| PD-related symptoms | 3 | 21 | 1 | 20 |
| Diarrhea | 3 | 21 | 0 | 20 |
| Diplopia | 3 | 21 | 0 | 20 |
| Back pain | 2 | 21 | 5 | 20 |
| Drug effect decreased | 2 | 21 | 4 | 20 |
| Head injury | 2 | 21 | 4 | 20 |
| Joint injury | 2 | 21 | 4 | 20 |
| Application site pain | 1 | 21 | 4 | 20 |
| Insomnia | 1 | 21 | 3 | 20 |
| Impulsive behaviour | 0 | 21 | 3 | 20 |

Table A9: Variable description

| **Variable name** | **Variable description** |
| --- | --- |
| adverse_events | Adverse event name |
| gdnf_n | number of events in GDNF arm |
| gdnf_N | total number of participants in GDNF arm |
| placebo_n | number of events in placebo arm |
| placebo_N | total number of participants in placebo arm |

Table A10: Code required to produce the volcano and dot plot in figure 2 using data from table A8

| **Stata code** | **Description** |
| --- | --- |
| - use dataset_name.dta, clear | Call dataset name saved data from table A8 |
| - drop if adverse_events =="Patients with at least one TEAE " | Drop the observation that summarises overall number of AEs |
| - aevolcs adverse_events, n1(gdnf_n) n2(placebo_n) tot1(gdnf_N) tot2(placebo_N) labelyn(1) label(0.75) labang(0) labgap(3) labpos1(10) labpos2(1) yaxismax(0) legendyn(1) legend1(Increased risk in GDNF) legend2(Increased risk in Placebo) | Command to produce volcano plot. Options n1, n2, tot1 and tot1 required. Other options used to format figure. |
| - aedots event, n1(gdnf_n) n2(placebo_n) tot1(gdnf_N) tot2(placebo_N) leftsymb1(triangle) legendleftyn(1) logoff(1) rightxline(1) rightxlabel(0.01 0.03 0.2 0.5 1 2 6 50 150 ) legendleft1("GDNF (N=21)") legendleft2("Placebo (N=20)") leftcolor1(red) leftcolor2(blue) trightmargin(2) nummargin(1) margin(25) event1pos(500) event2pos(2000) event1name(GDNF) event2name(Placebo) | Command to produce dot plot. Options n1, n2, tot1 and tot1 required. Other options used to format figure. |

**Case study 3: Example dataset of individual participant data**

Example datasets with n=30 unique intervention participants and n=31 unique placebo participants are provided when users downloaded the Stata commands aevolcano (example_volcano.dta) and aedot (example_dot.dta). We use example_volcano.dta to illustrate implementation of the Volcano and Dot plot when analysing individual participant data.

Table A11: Snapshot of dataset used to produce figure A3.

| usubjid | arm_name | arm | ae_pt | aebodsys | ae_ptnum | ae_bsnum |
| --- | --- | --- | --- | --- | --- | --- |
| 2001 | Placebo | 2 | Cold | Respiratory | Cold | Respiratory |
| 2001 | Placebo | 2 | Anemia | Blood and lymphatic | Anemia | Blood and lymphatic |
| 2001 | Placebo | 2 | Anemia | Blood and lymphatic | Anemia | Blood and lymphatic |
| 2001 | Placebo | 2 | Leukocytosis | Blood and lymphatic | Leukocytosis | Blood and lymphatic |
| 2001 | Placebo | 2 | Nausea | Gastrointestinal | Nausea | Gastrointestinal |
| 2001 | Placebo | 2 | Cold | Respiratory | Cold | Respiratory |
| 2001 | Placebo | 2 | Nausea | Gastrointestinal | Nausea | Gastrointestinal |
| 2001 | Placebo | 2 | Proteinuria | Renal and urinary | Proteinuria | Renal and urinary |
| 2001 | Placebo | 2 | Anxiety | Psychiatric | Anxiety | Psychiatric |
| 2001 | Placebo | 2 | Vomiting | Gastrointestinal | Vomiting | Gastrointestinal |
| 2001 | Placebo | 2 | Anemia | Blood and lymphatic | Anemia | Blood and lymphatic |
| 2002 | Intervention | 1 | Other | Blood and lymphatic | Other | Blood and lymphatic |
| 2002 | Intervention | 1 | Toothache | Other | Toothache | Other |
| 2002 | Intervention | 1 | Accident | Other | Accident | Other |
| 2004 | Placebo | 2 | Cystitis | Renal and urinary | Cystitis | Renal and urinary |
| 2004 | Placebo | 2 | Vomiting | Gastrointestinal | Vomiting | Gastrointestinal |
| 2004 | Placebo | 2 | Vomiting | Gastrointestinal | Vomiting | Gastrointestinal |
| 2004 | Placebo | 2 | Vomiting | Gastrointestinal | Vomiting | Gastrointestinal |
| 2004 | Placebo | 2 | Anemia | Blood and lymphatic | Anemia | Blood and lymphatic |
| 2004 | Placebo | 2 | Dry skin | Dermatological | Dry skin | Dermatological |
| 2004 | Placebo | 2 | Acid reflux | Gastrointestinal | Acid reflux | Gastrointestinal |
| 2004 | Placebo | 2 | Vomiting | Gastrointestinal | Vomiting | Gastrointestinal |
| 2004 | Placebo | 2 | Photosensitivity | Dermatological | Photosensitivity | Dermatological |
| 2005 | Placebo | 2 | Anemia | Blood and lymphatic | Anemia | Blood and lymphatic |
| 2005 | Placebo | 2 | Anemia | Blood and lymphatic | Anemia | Blood and lymphatic |
| 2005 | Placebo | 2 | Anemia | Blood and lymphatic | Anemia | Blood and lymphatic |
| 2007 | Placebo | 2 | Abdominal pain | Gastrointestinal | Abdominal pain | Gastrointestinal |
| 2007 | Placebo | 2 | Anxiety | Psychiatric | Anxiety | Psychiatric |
| 2007 | Placebo | 2 | Headache | Neurological | Headache | Neurological |
| 2007 | Placebo | 2 | Headache | Neurological | Headache | Neurological |
| 2007 | Placebo | 2 | Anemia | Blood and lymphatic | Anemia | Blood and lymphatic |
| 2007 | Placebo | 2 | Anemia | Blood and lymphatic | Anemia | Blood and lymphatic |
| 2007 | Placebo | 2 | Pruritus | Dermatological | Pruritus | Dermatological |
| 2007 | Placebo | 2 | Sinus pain | Neurological | Sinus pain | Neurological |
| 2007 | Placebo | 2 | Anxiety | Psychiatric | Anxiety | Psychiatric |
| 2007 | Placebo | 2 | Bloating | Gastrointestinal | Bloating | Gastrointestinal |
| 2007 | Placebo | 2 | Anemia | Blood and lymphatic | Anemia | Blood and lymphatic |
| 2007 | Placebo | 2 | Anemia | Blood and lymphatic | Anemia | Blood and lymphatic |
| 2008 | Intervention | 1 | Other | Blood and lymphatic | Other | Blood and lymphatic |
| 2008 | Intervention | 1 | Anemia | Blood and lymphatic | Anemia | Blood and lymphatic |
| 2008 | Intervention | 1 | Colitis | Gastrointestinal | Colitis | Gastrointestinal |
| 2008 | Intervention | 1 | Anemia | Blood and lymphatic | Anemia | Blood and lymphatic |
| 2008 | Intervention | 1 | Anemia | Blood and lymphatic | Anemia | Blood and lymphatic |
| 2008 | Intervention | 1 | Colitis | Gastrointestinal | Colitis | Gastrointestinal |
| 2009 | Intervention | 1 | Anxiety | Psychiatric | Anxiety | Psychiatric |
| 2010 | Intervention | 1 | Anemia | Blood and lymphatic | Anemia | Blood and lymphatic |

Table A12: Variable description

| **Variable name** | **Variable description** |
| --- | --- |
| usubjid | Unique participant identifier |
| arm_name | Treatment group name |
| arm | Treatment group number (1:Intervention; 2:Placebo) |
| ae_pt | Adverse event name at preferred term level |
| aebodsys | Adverse event name at body system level |
| ae_ptnum | Adverse event name at preferred term level, numerical coding |
| ae_bsnum | Adverse event name at body system level, numerical coding |

Table A13: Code required to produce the volcano and dot plot in figure A3 using data from table A11

| **Stata code** | **Description** |
| --- | --- |
| - use example_volcano.dta, clear | Call example dataset. |
| - aevolcano aebodsys, treat(arm) id(usubjid) n1(30) n2(31) legendyn(1) legend1(Increased risk in Intervention ) legend2(Increased risk in Placebo) yaxismax(0.2) labpos(3) xaxismin(-0.1) xaxismax(0.2) labelyn(1) labang(0) | Command to produce volcano plot. Options treat, id, n1 and n2 required. Other options used to format figure. |
| - aedot aebodsys, treat(arm) id(usubjid) n1(30) n2(31) leftsymb1(triangle) leftcolor1(red) leftcolor2(blue) legendleft1(Intervention (N=31)) legendleft2(Placebo (N=30)) brightmargin(7) trightmargin(8) logoff(1) rightxline(1) rightxlabel( 0.03 0.2 0.5 1 2 6 50 ) nummargin(1) margin(35) event1pos(90) event2pos(500) event1name(Intervention) event2name(Placebo) | Command to produce dot plot. Options treat, id, n1 and n2 required. Other options used to format figure. |


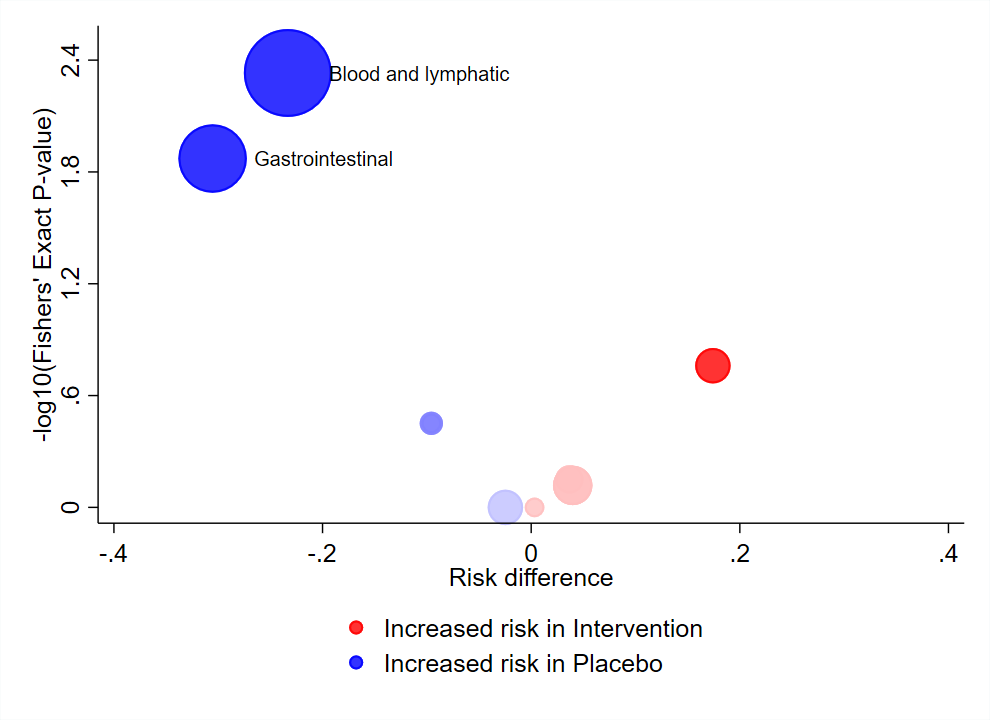


**
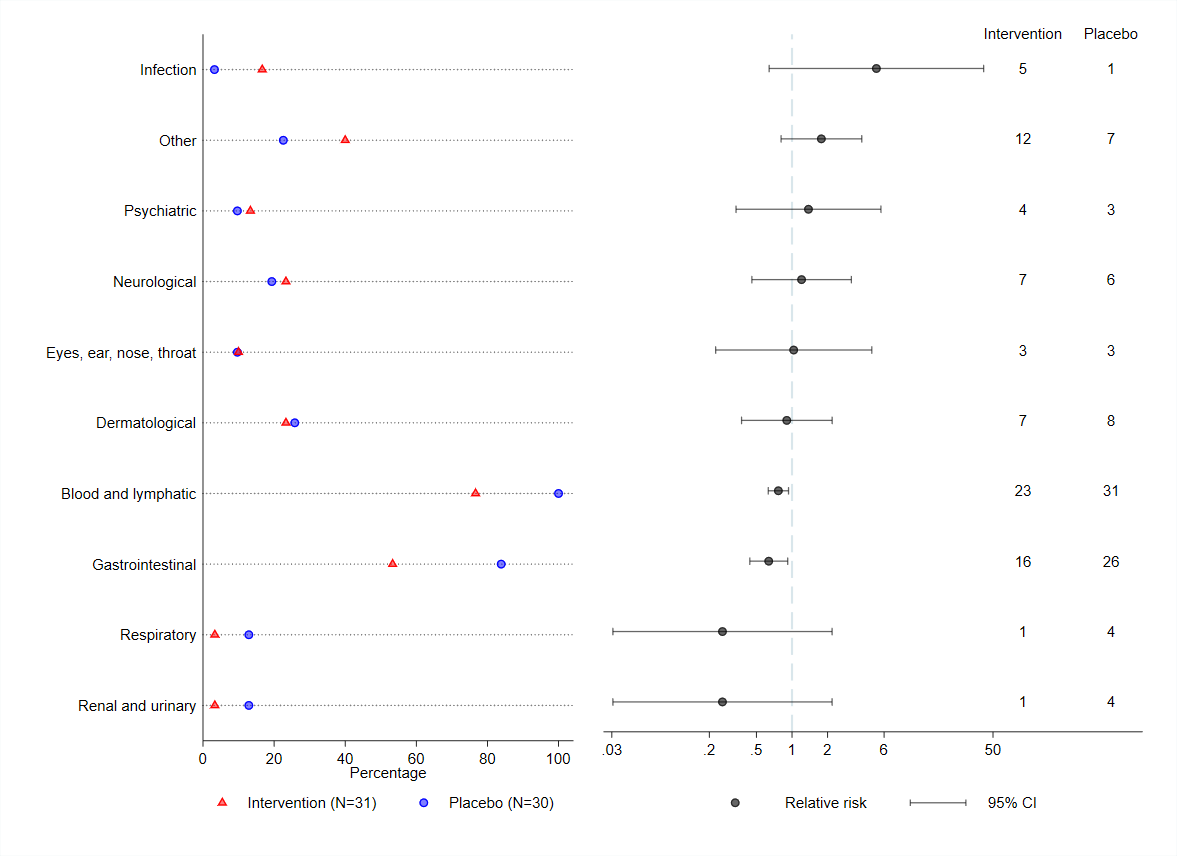
**

**Figure A3a:** Volcano Plot for **adverse events** between two treatment arms from an example dataset of individual participant data. *The x-axis represents the difference in proportions of participants experiencing each adverse event between the treatment arms (intervention – placebo). The y-axis represents the p-value from a Fisher’s exact test on the -log10 scale. The centre of the bubble indicates the coordinates for each adverse event. The size of the bubble is proportional to the total number of events for both treatment groups combined. Colour is used to indicate direction of treatment effect with red indicating greater risk in the intervention group and blue indicating greater risk in the placebo group. Colour saturation corresponds to the -log10(p-value) for each event.*

**Figure A3b:** Dot Plot for **adverse events** between two treatment arms from an example dataset of individual participant data. *The left side of the figure displays the percentage of participants experiencing an adverse event (labelled on the y-axis) in the intervention group with a red triangle and placebo group with a blue circle. The right side of the figure displays the relative risk and corresponding 95% confidence interval on the log10 scale.*
